# Digital mental health interventions as stand-alone vs. augmented treatment as usual

**DOI:** 10.1186/s12889-024-18412-1

**Published:** 2024-04-05

**Authors:** Benjamin W. Nelson, Nicholas C. Peiper, Valerie L. Forman-Hoffman

**Affiliations:** 1 Meru Health Inc, 19 South B Street, Ste 3, 94401 San Mateo, CA USA; 2 Department of Psychology, University of North Carolina at Chapel Hill, 235 E. Cameron Avenue, 27599 Chapel Hill, NC USA; 3 Department of Epidemiology and Population Health, University of Louisville, 2314 S. Floyd Street, 40292 Louisville, KY USA; 4 Department of Epidemiology, The University of Iowa, 52242 Iowa City, IA USA

**Keywords:** Anxiety, Depression, Digital mental health, Smartphone

## Abstract

**Background:**

Smartphone-based digital mental health interventions (DMHI) have been described as a purported solution to meet growing healthcare demands and lack of providers, but studies often don’t account for whether patients are concurrently in another treatment modality.

**Methods:**

This preregistered quasi-experimental intent-to-treat study with 354 patients enrolled in a therapist-supported DMHI examined the treatment effectiveness of the Meru Health Program (MHP) as a stand-alone treatment as compared to the MHP in combination with any other form of treatment, including (1) in-person therapy, (2) psychotropic medication use, and (3) in-person therapy and psychotropic medication use.

**Results:**

Patients with higher baseline depressive and anxiety symptoms were more likely to self-select into multiple forms of treatment, an effect driven by patients in the MHP as adjunctive treatment to in-person therapy and psychotropic medication. Patients in combined treatments had significantly higher depressive and anxiety symptoms across treatment, but all treatment groups had similar decreasing depressive and anxiety symptom trajectories. Exploratory analyses revealed differential treatment outcomes across treatment combinations. Patients in the MHP in combination with another treatment had higher rates of major depressive episodes, psychiatric hospitalization, and attempted death by suicide at baseline.

**Conclusions:**

Patients with higher depressive and anxiety symptoms tend to self-select into using DMHI in addition to more traditional types of treatment, rather than as a stand-alone intervention, and have more severe clinical characteristics. The use the MHP alone was associated with improvement at a similar rate to those with higher baseline symptoms who are in traditional treatments and use MHP adjunctively.

## Introduction

Over the last decade there has been a growing literature examining the effectiveness of smartphone-based digital mental health interventions (DMHI) for various mental health needs. Recent randomized control trials, systematic reviews, and stand-alone studies have found that DMHIs can be effective in reducing depression and anxiety symptoms [1–5] and improving wellbeing [5], even for patients reporting moderately-severe to severe symptoms [2]. While these interventions have enabled the provision of evidence-informed treatment at scale to meet growing mental healthcare demands that have been further compounded by the scarcity of providers, outcomes studies of DMHIs have often not accounted for whether patients are concurrently using another treatment modality, such as in-person therapy, psychotropic medication use, or a combination of these interventions. When data on concurrent treatment types are collected, these variables are typically used as covariates to adjust efficacy or effectiveness analyses, rather than to stratify patients into treatment type groups. For example, a recent study found that a DMHI was associated with significant reductions in moderately-severe to severe depressive symptoms, but that the decline in symptoms was greater for those also taking psychotropic medications [2]. The omission of stratifying patients by combination of treatment types when combined with a lack of large-scale randomized control trials (RCTs) with active controls has the potential to inflate treatment effects in observational studies [6]. Nevertheless, leveraging the existence of natural experiments may yield informative results about comparative treatment effects that emulate those in RCTs and provide robust base rates for further investigation in RCTs [7–10]. This brief report examined the comparative effectiveness of the Meru Health Program (MHP) as a stand-alone treatment as compared to the MHP in combination with any additional treatment. We then expand our analyses comparing the MHP as a stand-alone treatment to three treatment combinations, including (1) in-person therapy, (2) psychotropic medication use, and (3) in-person therapy and psychotropic medication use.

### Current study

This intent-to-treat (ITT) study, preregistered on Open Science Framework (https://osf.io/r7z4y/), was devised to examine the comparative effectiveness of the MHP as a stand-alone treatment as compared to the MHP in combination with treatment as usual (two-arm). We probed these analyses with a four-arm design that compared (1) MHP only to the MHP as a combined treatment with (2) in-person therapy, (3) psychotropic medication use, or (4) both in-person therapy and psychotropic medication use. First, we hypothesized that patients with higher depressive and anxiety symptoms would self-select into using the MHP as an adjunctive treatment to another form of treatment, rather than as a stand-alone treatment, such that patients in the MHP only condition would have significantly lower baseline depressive and anxiety scores when compared to patients in the MHP as adjunctive treatment to any additional form of treatment (in-person therapy, psychotropic medication use, or both in-person therapy and psychotropic medication use). Second, we hypothesized that MHP in combination with any other treatment would moderate the effect of treatment week on depressive and anxiety symptom trajectory, such that patients in the MHP in combination with another treatment modality would have steeper decreases in depressive and anxiety symptoms when compared to MHP only.

## Methods and materials

### Participants and recruitment

Data came from 354 patients, aged 18–66 (see Table 1) recruited remotely in the United States and who started the MHP on or after 10/11/2021, when data on type(s) of other treatment started to be collected, and who ended treatment on or before 02/15/2022. Patients examined in this study came from two main referral streams, with some referred by healthcare providers and others gaining access to the MHP via an employee health, with varying out-of-pocket expense to enroll. Patients consented to participate and have their collected and deidentified data used for research purposes when accepting the MHP privacy practices. Data collected as part of care is stored in Health Insurance Portability and Accountability Act-compliant electronic medical records that includes protected health information. All data are encrypted in transit, and at rest. Institutional review board exemption for this analysis was obtained from the Pearl Institutional Review Board (21-Meru-114) for analyses of previously collected and de-identified data. Inclusion/exclusion criteria of the MHP required patients to be at least 18 years of age or older, have at least mild levels of self-reported depression, anxiety, or burnout, own a smartphone, and not have an active substance use disorder, severe active suicidal ideation with a specific plan, severe active self-harm, or a history of psychosis or mania.

**Table 1:** Patient demographics and treatment outcomes

Variable		N (Percent)	Mean (SD), Range
Patient Demographics			
Age			38.1 (10.6), 18–66
Gender	Male	77 (21.75%)	
	Female	276 (77.97%)	
	Non-Binary	1 (0.28%)	
Race	White	276 (77.97%)	
	Black	13 (3.67%)	
	Asian	64 (18.08%)	
	Other	1 (0.28%)	
Treatments			
	MHP Only	150 (42.37%)	
	MHP and In Person Therapy	100 (28.25%)	
	MHP and Psychotropic Medication Use	40 (11.30%)	
	MHP and both In Person Therapy and Psychotropic Medication Use	64 (18.08%)	
Baseline PHQ-7 Severity	Minimal	34 (9.60%)	
	Mild	107 (30.23%)	
	Moderate	95 (26.84%)	
	Moderately Severe	73 (20.62%)	
	Severe	45 (12.71%)	
Baseline GAD-7 Severity	Minimal	14 (3.95%)	
	Mild	106 (29.94%)	
	Moderate	130 (36.72%)	
	Severe	104 (29.38%)	

### Meru health program (MHP) treatment

The MHP has been described in detail in prior publications [11]. In sum, the MHP is a 12-week, therapist-supported treatment composed of several different components of evidence-informed and emerging new therapies delivered via a smartphone app [12–18].

#### Measures

All measures examined in this study were collected in the MHP app at baseline and biweekly during the MHP including at end-of-treatment.

##### Patient health questionnaire-9 (PHQ-9)

Patients completed the PHQ-9 to screen for depression [19]. The PHQ-9 has excellent internal consistency (Cronbach’s α = 0.89 in primary care settings), and excellent test-retest reliability (ICC = 0.84) [19].

##### Generalized anxiety disorder-7 (GAD-7)

Patients completed the GAD-7 to screen for anxiety [20]. The GAD-7 has excellent internal consistency (Cronbach’s α = 0.92), and excellent test-retest reliability (ICC = 0.83) [20].

##### Mental health treatment

During the intake questionnaire, patients completed the following question, “Are you planning to receive counseling or therapy from a mental health professional, such as a psychologist, psychiatrist, or clinical social worker during your participation in the Meru Health Program?” with the option to select “Yes” or “No”.

##### Medication

“I currently take prescribed medication for a mental health condition.” with an option to select a checkbox.

##### Treatment Outcomes

- Minimally Clinically Important Difference (MCID) refers to smallest change in scores that are of “perceived benefit” to the patient and was defined as a PHQ-9 score reduction of ≥ 5 [21] and a GAD-7 score reduction of ≥ 4 [22] for patients with a 5 or above starting PHQ-9 and GAD-7 score, respectively.
- Clinically Significant Improvement (CSI) refers to a PHQ-9 score less than 10 and a 50% decline from the pretreatment score [2, 19]. Due to lack of clear definition for the GAD-7, we used the same definition as PHQ-9.
- Remission was defined as an end-of-program PHQ-9 < 5 [23] for patients with baseline PHQ-9 of 5 or above. Like the CSI definition above, due to a lack of a clear definition for remission for the GAD-7 we used the same definition of an end-of-program GAD-7 < 5 for patients with a 5 or above GAD-7 at baseline.
- Dropout was defined as any patient that did not complete at least one practice each week during the first 6 weeks of treatment.

##### Covariates

Participant age, gender, race (all hypotheses), and dropout status (hypothesis 2) were used as covariates.

### Statistical analysis

All statistical analyses were conducted with RStudio, Version 1.3.959. Statistical significance was defined using *p*-values at the 0.05 threshold. Descriptive statistics were calculated for each patient demographic and clinical variable (see Table 1). Outcome measures were analyzed using ITT analysis in which all participants with outcome measures at baseline were included, regardless of intervention engagement or attrition by using multiple imputation with the mice package [24].

First, we examined the impact of stand-alone MHP as compared to MHP as adjunctive to any other of the three different treatment combinations (0- MHP stand-alone vs. 1- MHP as adjunctive to any additional form of care) on baseline PHQ-9 and GAD-7 symptom severity by using an ANCOVA with the car package [25]. Next, we ran a post hoc ANCOVA to examine whether there were significant differences between the MHP and those in the MHP as adjunctive to each additional form of care (0- MHP stand-alone, 1- MHP as adjunctive to therapy, 2- MHP as adjunctive to psychotropic medication use, 3- MHP as adjunctive to in person therapy and psychotropic medication use). We then used the Tukey Method to adjust *p*-values for posthoc contrasts for multiple comparisons.

Second, in two separate models, we examined the interaction of treatment week and whether patients used the MHP as a stand-alone rather than as adjunctive to any one or more of the other treatment types (0- MHP stand-alone vs. 1- MHP as adjunctive to any additional form of care) on PHQ-9 and GAD-7 trajectory by conducting a multilevel model (MLM) using the lmer package [26] that nests week of treatment (Level-1) within individuals (Level-2). Fixed effects including covariates and whether patients are in another form of treatment, which were tested at the level of participants (Level-2). This statistical approach accounts for dependency within participants [27, 28]. We included a cross-level interaction with whether patients were in another form of treatment as a moderator of the association between treatment week and PHQ-9 and GAD-7 scores. We then ran a follow-up model to examine whether there are significant interactions between treatment week and whether patients were in the MHP alone or as adjunctive to each additional form of care (0- MHP stand-alone, 1- MHP as adjunctive to therapy, 2- MHP as adjunctive to psychotropic medication use, 3- MHP as adjunctive to in person therapy and psychotropic medication use).

Lastly, in exploratory analyses we examined whether the 4-group variable, MHP only as compared to MHP in combination with each additional treatment, had differential impacts on treatment outcomes including MCID, remission, and dropout by using chi-square tests using the ggstatsplot package [29] with MHP only as the reference. We also examined whether there were differences in key clinical characteristics, including number of major depressive episodes, history of psychiatric hospitalization, and history of attempted death by suicide using chi-square tests. Simulated data that preserves the structure of the initial data that support the findings of this study are available on request from Meru Health. The data are not publicly available due to containing sensitive clinical information that could compromise the privacy of research participants.

## Results

### Demographic and clinical characteristics

Table 1. presents patient demographic and clinical characteristics on the 354 patients included in this study. Most patients were female (77.97%) with an average age of 38.10 (standard deviation [SD] = 10.60). The mean baseline PHQ-9 was 12.00 (SD = 5.76) and the mean baseline GAD-7 was 11.80 (SD = 4.61), indicating moderate levels of depression and anxiety, respectively, which fall above the diagnostic cutoffs indicative of likely depression and anxiety diagnosis (Kroenke et al., 2001; Spitzer et al., 2006). Most patients reported being in another form of treatment while in the MHP, such that 42.37% were in the MHP stand-alone; 29.25% in the MHP and in-person therapy; 11.30% in the MHP and psychotropic medication use; and 18.08% in the MHP with in-person therapy and psychotropic medication use. Table 2. presents patient clinical characteristics by combination of treatment and indicate an overall greater proportion of those with a prior psychiatric hospitalization, *X*^2^ (3, *N* = 354) = 10.00, *p* = 0.02; suicide attempt, *X*^2^ (3, *N* = 354) = 10.00, *p* = 0.02; or greater number of prior major depressive disorder episodes, *X*^2^ (3, *N* = 354) = 31.00, *p* < 0.001, in patients that self-selected into multiple forms of treatment.

**Table 2:** Patient clinical characteristics by treatment combination

Clinical Characteristics	Treatment Combination	Frequencies							
MDD Episodes	Treatment Combination	No MDD Episodes		One MDD Episode		Two MDD Episodes		Two or More MDD Episodes	
	MHP	69	46.00%	33	22.00%	21	14.00%	26	17.33%
	MHP and Therapy	49	49.00%	17	17.00%	13	13.00%	21	21.00%
	MHP and Meds	14	35.00%	3	7.50%	7	17.50%	16	40.00%
	MHP, Therapy, and Meds	13	20.31%	16	25.00%	7	10.94%	28	43.75%
Psychiatric Hospitalization	Treatment Combination	None		Present					
	MHP	147	98.00%	2	1.33%				
	MHP and Therapy	91	91.00%	9	9.00%				
	MHP and Meds	36	90.00%	4	10.00%				
	MHP, Therapy, and Meds	58	90.63%	6	9.38%				
Suicide Attempt	Treatment Combination	None		Present					
	MHP	145	96.67%	4	2.67%				
	MHP and Therapy	96	96.00%	4	4.00%				
	MHP and Meds	36	90.00%	4	10.00%				
	MHP, Therapy, and Meds	56	87.50%	8	12.50%				

### Treatment combination and baseline symptom severity

#### PHQ-9

There was a significant difference in baseline PHQ-9 scores (f [1] = 10.29, *p* = 0.002), such that patients had significantly higher baseline PHQ-9 scores if they used MHP as an adjunct to any other treatment (Mean = 12.7, SD = 5.68) as compared to MHP only (Mean = 11.0, SD = 5.74) after controlling for age, gender, and race. Follow-up post-hoc analyses to examine each of the four specific treatment combinations had a significant main effect of treatment type (f [3] = 6.09, *p* = 0.001). Post-hoc contrasts with *p*-values adjusted with the Tukey Method for multiple comparisons found that this effect was driven by patients in the MHP in combination with in-person therapy and psychotropic medication use (Mean = 14.4, SD = 5.62) as compared to MHP only (Mean = 11.0, SD = 5.74).

#### GAD-7

There was a significant difference in baseline GAD-7 scores (f [1] = 4.14, *p* = 0.043), such that patients had significantly higher baseline GAD-7 scores if they used MHP as an adjunct to any other form of treatment (Mean = 12.2, SD = 4.69) as compared to MHP only (Mean = 11.2, SD = 4.45). Follow-up post-hoc analyses to examine each of the four specific treatment combinations was not significant for the main effect of treatment type (f [3] = 2.54, *p* = 0.057).

### Depressive and anxiety symptom trajectories by treatment combination

#### PHQ-9

Treatment week was significantly and inversely associated with PHQ-9 scores (beta = -0.454, SE = 0.032, *p* < 0.001). Patients that were in MHP as an adjunct to any other form of treatment had significantly higher PHQ-9 scores across treatment (beta = 1.522, SE = 0.564, *p* = 0.007) when compared to the MHP as stand-alone, but there was no interaction between treatment type and treatment week (beta = -0.036, SE = 0.042, *p* = 0.394), indicating that both treatment groups had similar symptom trajectories after controlling for age, gender, race, and completion status. Follow-up analyses to examine each of the four specific treatment combinations indicated that the treatment effect was driven by patients in the MHP as adjunctive treatment to both in-person therapy and psychotropic medication use, such that these patients had significantly higher PHQ-9 scores across treatment as compared to patients in the MHP only group (beta = 3.181, SE = 0.774, *p* < 0.001) and again there were no interaction between each treatment combination and treatment week (*p* > 0.05).

#### GAD-7

Treatment week was significantly and inversely associated with GAD-7 scores (beta = -0.450, SE = 0.027, *p* < 0.001). Patients that were in MHP as an adjunct to any other form of treatment had significantly higher GAD-7 scores across treatment (beta = 0.983, SE = 0.448, *p* = 0.028), but there was no interaction between treatment combination and treatment week (beta = -0.034, SE = 0.035, *p* = 0.332), indicating that both treatment groups had similar symptom trajectories after controlling for age, gender, race, and completion status. Follow-up analyses to examine each of the four specific treatment combinations indicated that the treatment effect was driven by patients in the MHP as an adjunct to both in-person therapy and psychotropic medication use, such that these patients had significantly higher GAD-7 scores across treatment as compared to patients in the MHP only group (beta = 1.861, SE = 0.621, *p* = 0.003) and again there were no interaction between each treatment combination and treatment week (*p* > 0.05).

### Exploratory analyses

We used a series of chi-square tests that were supplemented by frequency tables to examine treatment outcome proportions of patients having end-of-treatment MCID, remission, and dropout for each of the 4 treatment groups without adjusting for covariates.

#### MCID

**Fig. 1:**
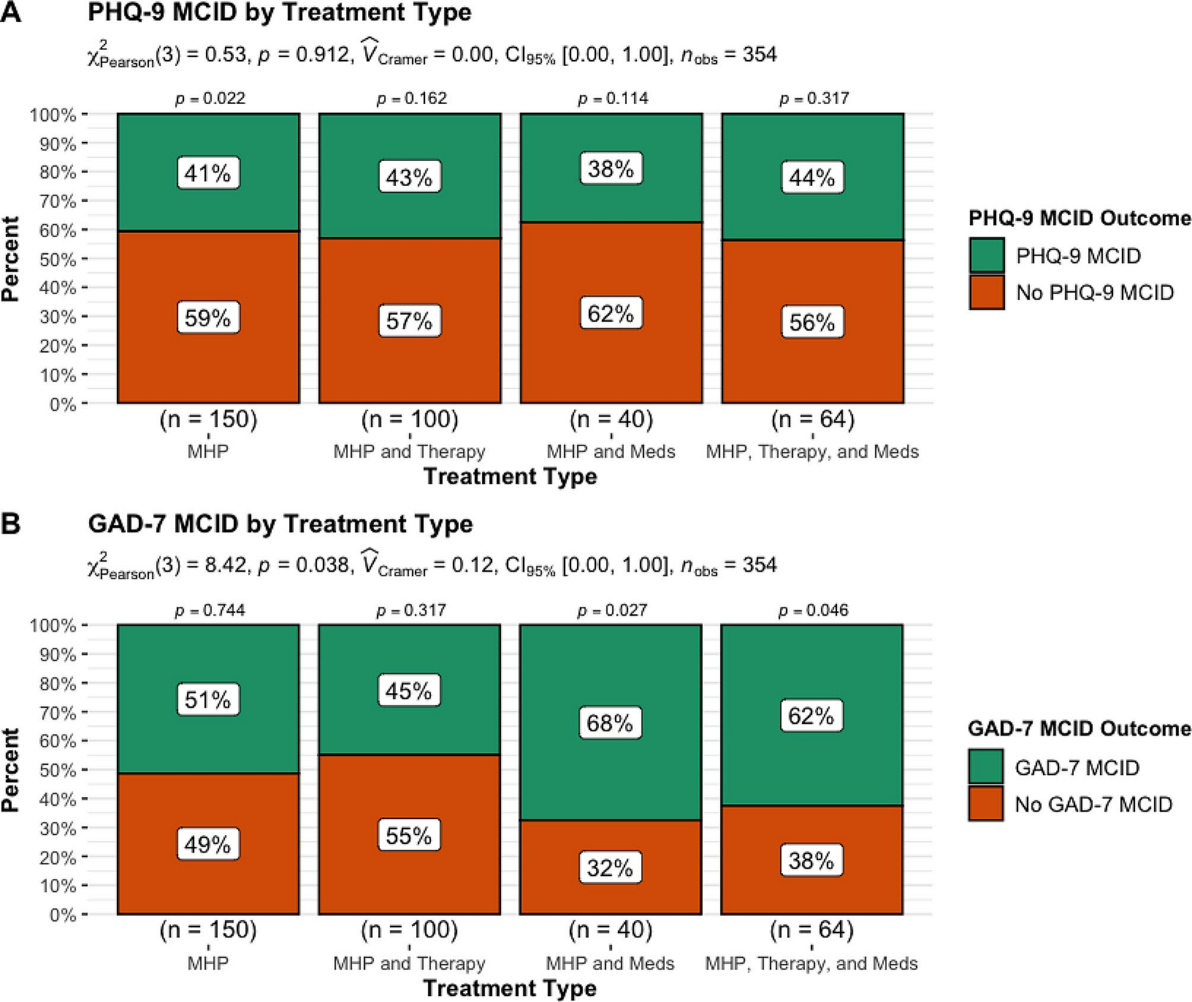
PHQ-9 (**A**) and GAD-7 (**B**) MCID outcome by treatment type Note: The subtitle displays the Pearson’s chi-squared test, Cramer’s V effect size, 95% confidence interval and number of observations. The *p*-value above each treatment type is the proportion test examining whether the proportion for the treatment outcome is significant for each treatment type

When examining proportions of MCID, there were no group differences in PHQ-9 MCID, *X*^2^ (3, *N* = 354) = 0.53, *p* = 0.91, but there were significant group differences for GAD-7 MCID, *X*^2^ (3, *N* = 354) = 8.42, *p* = 0.038 (see Fig. 1). Furthermore, pairwise comparisons showed that for the MHP as a stand-alone treatment there were significantly more patients that did not reach PHQ-9 MCID as compared to those that did reach PHQ-9 MCID (*p* = 0.022), while there were no pairwise differences in PHQ-9 MCID for any other treatment (*p* > 0.05). In contrast, when examining pairwise comparisons for GAD-7 MCID, patients in the MHP in combination with psychotropic medication (*p* = 0.027) and MHP in combination with both in-person therapy and psychotropic medication (*p* = 0.046) had greater levels of MCID, while there were no significant differences for other treatment combinations (*p* > 0.05).

#### Remission

**Fig. 2:**
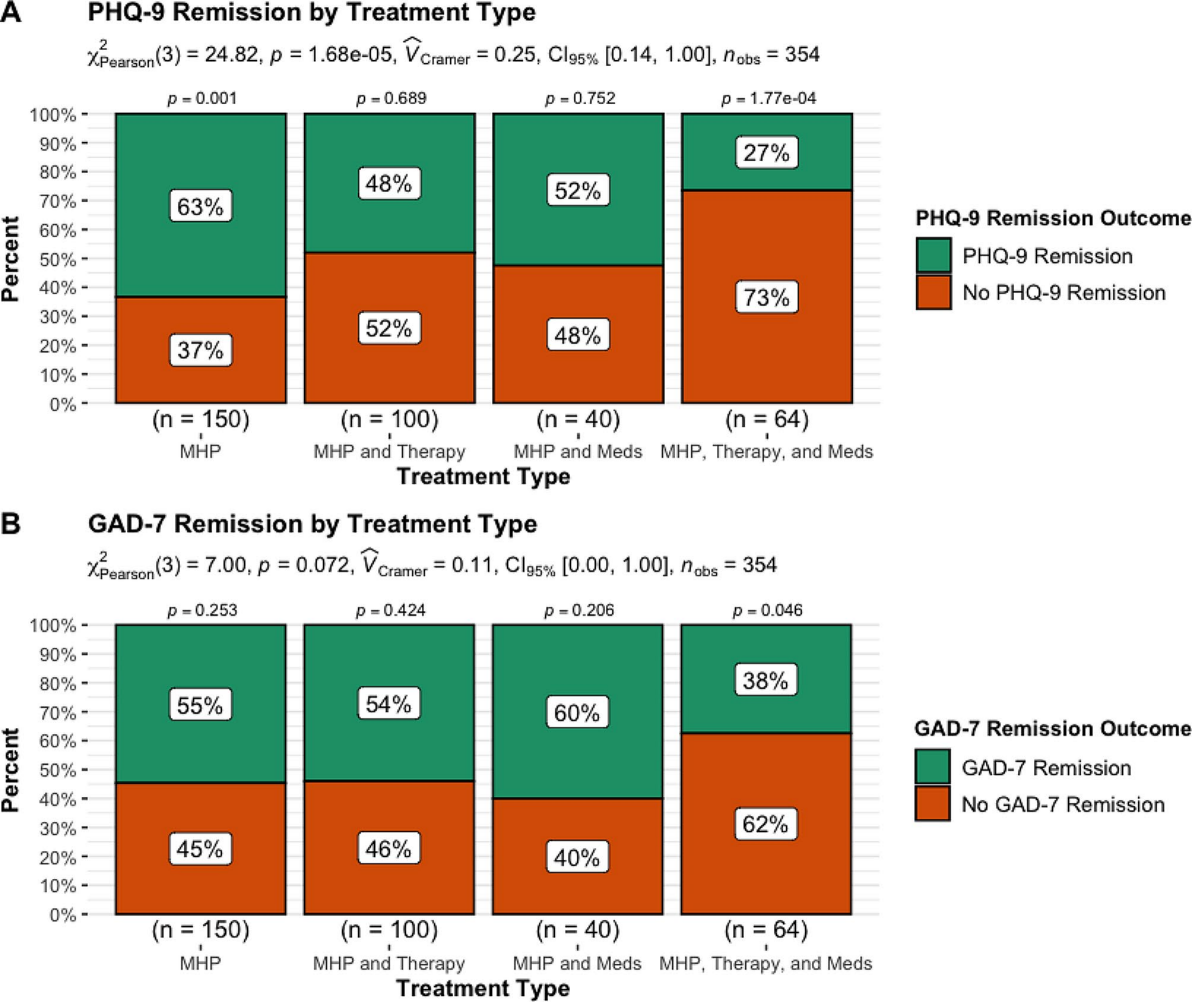
PHQ-9 (**A**) and GAD-7 (**B**) remission outcome by treatment type Note: The subtitle displays the Pearson’s chi-squared test, Cramer’s V effect size, 95% confidence interval and number of observations. The *p*-value above each treatment type is the proportion test examining whether the proportion for the treatment outcome is significant for each treatment type

When examining proportions of remission, there were significant differences for PHQ-9 remission, *X*^2^ (3, *N* = 354) = 24.82, *p* = 0.001, but there were no group differences for GAD-7 remission, *X*^2^ (3, *N* = 354) = 7.00, *p* = 0.07 (see Fig. 2). Furthermore, pairwise comparisons showed that for the MHP as a stand-alone treatment there were significantly more patients that reached PHQ-9 remission (*p* = 0.001) as compared to those that did not and for those in the MHP in combination with both in-person therapy and psychotropic medication use there was significantly less PHQ-9 remission (*p* < 0.001), while there was no significant difference in PHQ-9 remission for the other two treatment groups (*p* > 0.05). In addition, when examining rates of GAD-7 remission there were no significant differences in remission rates within each treatment type (*p* > 0.05), except for those in the MHP as an adjunct to both in-person therapy and psychotropic medication use, such that there were significantly more patients in this group that did not reach remission as compared to patients that did reach remission (*p* = 0.046).

#### Dropout

**Fig. 3:**
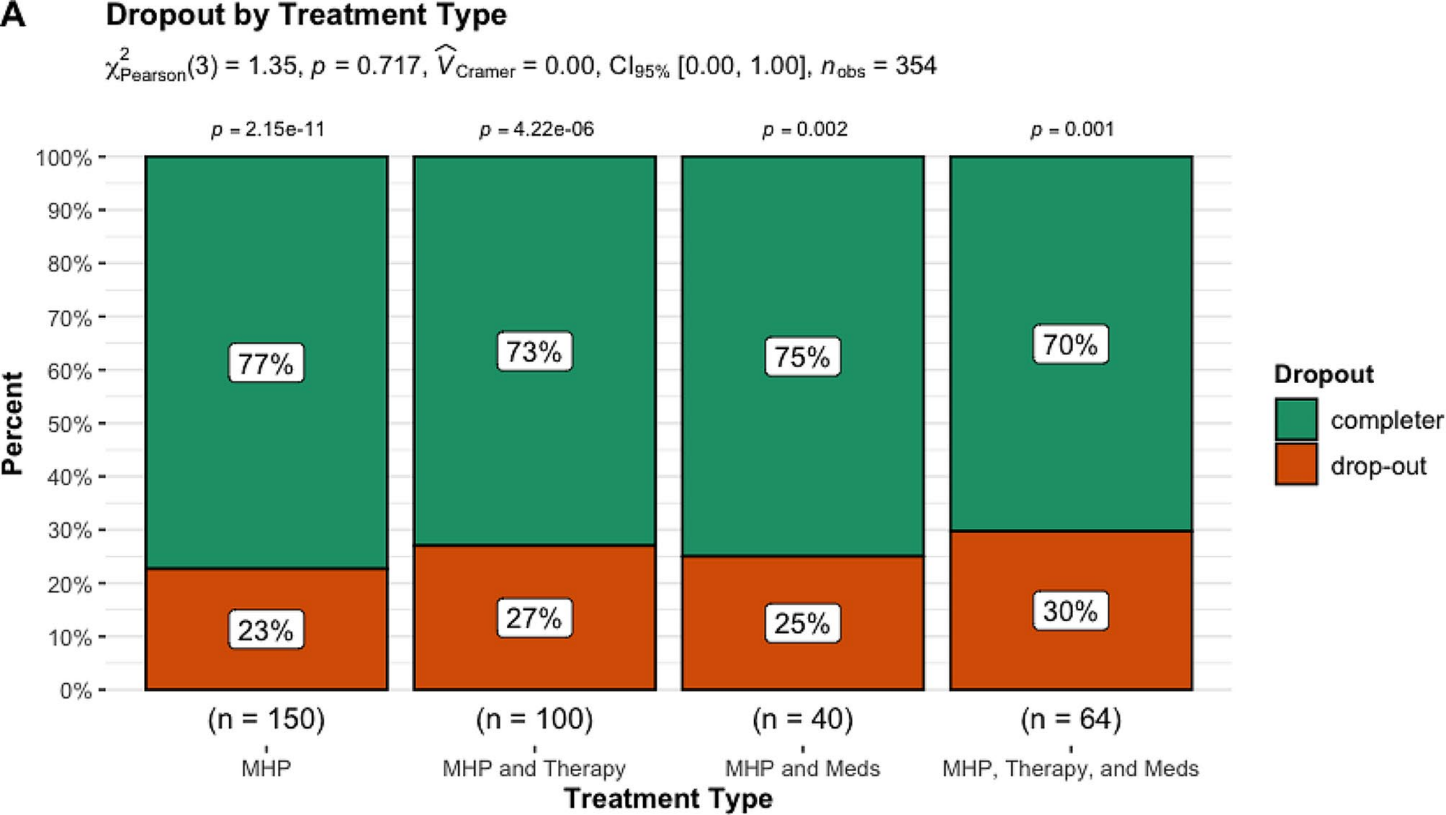
Dropout by treatment type Note: The subtitle displays the Pearson’s chi-squared test, Cramer’s V effect size, 95% confidence interval and number of observations. The *p*-value above each treatment type is the proportion test examining whether the proportion for the treatment outcome is significant for each treatment type

When examining rates of dropout, there were no group differences, *X*^2^ (3, *N* = 354) = 1.35, *p* = 0.72 (see Fig. 3). Furthermore, when examining pairwise comparisons all treatments had significantly more patients complete the MHP than not (*p* < 0.01).

## Discussion

This preregistered quasi-experimental ITT study was devised to interrogate the difference in treatment effectiveness of the MHP as a stand-alone treatment as compared to the MHP in combination with any type of treatment as usual. In addition, this study followed up on these findings by dismantling treatment combinations to compare the MHP as a stand-alone treatment to the MHP as an adjunctive treatment to (1) in-person therapy, (2) psychotropic medication use, and (3) in-person therapy and psychotropic medication use. Overall, results were partly consistent with hypotheses.

First, over half of patients presenting to the MHP were receiving some additional form of treatment. Patients that used the MHP as an adjunctive treatment to standard forms of care tended to have significantly greater clinically severe histories characterized by greater history of psychiatric hospitalization, attempted death by suicide, and greater numbers of past major depressive disorder episodes than those using the MHP as a stand-alone treatment.

In line with our hypotheses, patients that were receiving any additional form of treatment in combination with the MHP had significantly higher baseline depressive and anxiety symptom severity. This finding may indicate that patients with more severe symptoms may possibly self-select into traditional forms of care with the MHP used as an adjunct treatment as compared to patients with lower levels of symptoms, where the MHP may be perceived as sufficient for dealing with their level of symptoms. When we probed this treatment effect of the MHP as a stand-alone as compared the MHP as an adjunct to each of the three additional types of treatment with the four-arm design, we discovered that this effect was driven by patients that were in the MHP in combination with in-person therapy and psychotropic medication use (18% of patients; four-arm design). This indicates that the main effects found between MHP as a stand-alone as compared the MHP as an adjunct to any additional type of treatment were driven by the subsample of patients that were in the additional treatment group that received both in-person therapy and psychotropic medication.

When examining symptom trajectories across treatment, the finding of stratified depressive and anxiety symptom severity at baseline was replicated across treatment, such that patients in the MHP as an adjunct to any other form of treatment (two-arm design) had significantly higher depressive and anxiety symptoms throughout treatment. When we probed this effect using the four-arm design, we again replicated our baseline findings such that the effect by type of treatment was specifically driven by patients that were in the MHP in combination with in-person therapy and psychotropic medication use. In contrast to our hypotheses, there was no interaction effect between treatment week and type of treatment combination on depressive and anxiety symptoms across time, indicating that depressive and anxiety symptom trajectories (i.e., slopes) were similar between those using MHP as a stand-alone treatment as compared to those using MHP in combination with another treatment. This finding indicates that although patients started with significantly different severity of depressive and anxiety scores, those using MHP as a stand-alone treatment had similar depressive and anxiety symptom trajectories as compared with those who used the MHP as an adjunct to other treatment types. However, results from this study do not provide direct evidence for whether the MHP intervention was associated with treatment gains in those who are augmenting treatment as usual, because we did not collect data to identify what accounted for those treatment gains. While it may be possible that the MHP has utility among those with lower symptoms, but lacks an effect in a more severe population or it accounts for little-to-no effect, this is not likely to be the case as the MHP has been previously shown for those with moderately-severe to severe depressive symptoms to have overall improvements that were still significant even after adjusting for concurrent use of psychotropic medications, although the declines were larger for those using psychotropic medication [2]. In addition, using a repeated measures latent profile analysis, the MHP treatment has been found to show that (1) participants in a high-severity trajectory still experienced clinically significant improvements; (2) engagement was actually the highest among the high severity trajectory; and (3) that program completion was approximately 93% in patients with a high severity trajectory [3]. This finding indicates that if the program wasn’t working for high severity depression, then we would likely not observe these effects and would instead see rapid disengagement among patients with high rates of non-completion. Lastly, MHP finding showed that patients who disengaged quickly were less likely than other groups to report previous MDEs, psychiatric hospitalizations, and suicide attempts, indicating that this treatment is tolerated amongst patients with severe depressive symptoms [4]. It should also be noted that some prior literature has found that those with lower baseline symptoms are associated with treatment response [30], although faster improvement has been found for those with higher baseline symptom severity [31], indicating that this finding is mixed.

Lastly, when examining treatment outcomes between the MHP as a stand-alone treatment as compared to the MHP in combination with (1) in-person therapy, (2) psychotropic medication use, or (3) in-person therapy and psychotropic medication use, we found that in terms of depression symptom MCID there were no significant differences between groups, but when examining each treatment for rates of MCID, we found that significantly fewer patients in the MHP as a stand-alone reached MCID as compared to those that did not. In contrast, we did find that there was a significant difference for depressive symptom remission with significantly greater numbers of patients reaching remission within the MHP stand-alone than not reaching remission and significantly fewer patients reaching remission within the MHP as an adjunct to both in-person treatment and psychotropic medication use. These two later findings are likely driven by baseline effects, such that significantly lower starting depressive symptoms in the MHP as a stand-alone allowed for patients to quickly reach the definition for remission, while the significantly higher starting depressive symptoms for patients in the MHP as an adjunct to both in-person treatment and psychotropic medication use made it much harder for patients to reach remission.

In contrast to findings for depressive symptom treatment outcomes, results for anxiety symptom outcomes indicated that in terms of anxiety symptom MCID there were significant differences between treatment groups. When examining MCID within each treatment type, we found that for patients in the MHP as adjunct to psychotropic medication use and MHP as adjunct to both in-person therapy and psychotropic medication use significantly greater numbers of patients reached MCID. These two findings indicate that when treating anxiety symptoms, patients would likely benefit the most if the MHP is combined with psychotropic medication or both in-person therapy and psychotropic medication use. In contrast, to anxiety MCID findings, there were no significant differences for anxiety symptom remission, although when examining remission within each treatment type, we found that patients in the MHP as adjunct to both in-person therapy and psychotropic medication use had lower rates of remission. Lastly, there were no significant differences in dropout by treatment type, but there were significantly greater numbers of patients that completed treatment in each treatment type as compared to those that dropped out.

### Limitations and future directions

While this study had several significant strengths, including a large sample size and comparing MHP alone vs. in combination with other treatments, the results should be interpreted while considering several limitations. First, this study was not a formal RCT where patients were randomized to different treatment combinations to have the highest level of evidence, although this natural experiment design has recently been called for in the field and provides moderate evidence for differential treatment effectiveness of stand-alone smartphone treatments as compared to a combination of smartphone and traditional treatments [7–10]. Second, we did not collect data on the type of in-person treatment or examine the specific type of psychotropic medication use of patients, which prevented us from examining more specific combinations of treatment. Third, this patient population was largely White and Asian, indicating that other racial groups were much less likely to either have access to or opt into receiving this treatment. One of the purported strengths of digital mental health interventions is their ability to scale to provide treatment to diverse patient populations, but the current study was limited by the homogenous sample to be able to investigate this properly. Future studies with interventions that are disseminated more widely could examine who has access to digital mental health interventions, who selects into using these treatments, and how to make these interventions more accessible. Fourth, formal clinical intakes were not completed on patients that entered the MHP via referrals from employee assistance programs or via their healthcare provider, although PHQ-9 scores indicate that over 60% of participants had a likely diagnosis of depression. Future studies should make sure to do a formal clinical intake to provide a diagnosis at intake. Fifth, we did not collect information on patient socioeconomic status. Lastly, the measure for in-person psychotherapy captured intentions to engage with a mental health professional during the DMHI, although engagement was not confirmed.

### Clinical implications

Increased understanding of participant characteristics that influence whether patients choose to use DMHI as stand-alone treatment vs. as adjunctive treatment for psychotropic medication and psychotherapy may help provide context for when these novel interventions should be used. The findings that patients with higher depressive and anxiety symptoms tend to self-select into using DMHI in addition to more traditional types of treatment, rather than as a stand-alone intervention and have more severe clinical characteristics offers information that can be used to provide stepped models of care for patient populations looking to use DMHI in the future with a screening gate to indicate when patients should seek additional treatments with provided resources for local providers. Furthermore, DMHI may enhance patient care by providing ongoing support and mental health interventions for patients on waitlists, thereby maximizing health outcomes and patient readiness for treatment.

## Conclusion

Our findings demonstrate that patients with higher levels of baseline mental health symptoms tend to self-select into using DMHI in conjunction with in-person treatment and psychotropic medication for the management of their symptoms. In addition, the use the MHP alone was associated with improvement at a similar rate to those with higher baseline symptoms who are in traditional treatments and use MHP adjunctively. These results indicate that DMHI may play an important role in the stepped model of care for patients with minimal to moderate levels of depressive symptoms. Furthermore, DMHI can be leveraged while patients are on waitlists for psychotherapy or psychiatrist medication management and to provide behavioral skills as a supplement to traditional therapy in between weekly sessions. Future RCT that compare DMHI alone and in combination with traditional treatments will be necessary to more rigorously investigate comparative treatment effectiveness across the four possible treatment combinations in this particular DMHI.

## Data Availability

The datasets generated and analysed during the current study are not publicly available due containing sensitive clinical information that could compromise the privacy of research participants, but simulated data that preserves the structure of the initial data that support the findings of this study are available from the corresponding author on reasonable request.
